# Intravascular large B-cell lymphoma (IVLBCL) presenting with exclusive cutaneous involvement, no B-symptoms and negative PET-CT: A Case Study

**DOI:** 10.1016/j.lrr.2026.100619

**Published:** 2026-10-17

**Authors:** Juliana Gabzdilova, Atila David Homolya, Stefan Raffac, Frantisek Gmitter, Tomas Guman, Viktoria Hazikova, Martina Maria Raffacova, Filip Breznen

**Affiliations:** a Department of Hematology and Oncohematology, Louis Pasteur University Hospital, Faculty of Medicine, Pavol Jozef Šafárik University, Košice, Slovakia; b Department of Radiotherapy and Oncology, East Slovakia Institute of Oncology, Faculty of Medicine, Pavol Jozef Šafárik University, Košice, Slovakia; c Department of Clinical Immunology and Allergology, Louis Pasteur University Hospital and OKIA s.r.o, Košice, Slovakia; d Department of Pathology, Louis Pasteur University Hospital, Košice, Slovakia; e Department of Pharmacology, Faculty of Medicine, Pavol Jozef Šafárik University, Košice, Slovakia; f Faculty of Medicine, Pavol Jozef Šafárik University and OKIA s.r.o, Košice, Slovakia; g Department of Cardiology, Kardiocentrum AGEL-Šaca a.s., Faculty of Medicine, Pavol Jozef Šafárik University, Košice, Slovakia

**Keywords:** Intravascular large B-cell lymphoma, Cutaneous variant, Biopsy, R-CHOP Abbreviations, BCL-2, B-cell lymphoma 2, BCL-6, B-cell lymphoma 6, CD, cluster of differentiation, CNS, central nervous system, CSF, cerebrospinal fluid, DLBCL, diffuse large cell B-lymphoma, ENA, extractable nuclear antigen, FISH, fluorescence *in situ* hybridization, H&E, hematoxylin and eosin, HGB, hemoglobin, IPI, international prognostic index, IVLBCL, intravascular large B-cell lymphoma, LDH, lactate dehydrogenase, MCHC, mean corpuscular hemoglobin concentration, MCV, mean corpuscular volume, MUM1, multiple myeloma 1, NHL, non-hodgkin lymphoma, PET-CT, positron emission tomography, PLT, platelets, R-CHOP, rituximab, cyclophosphamide, doxorubicin, vincristine, prednisone, sIL-2R, soluble IL-2 receptor, SUVmax, maximal standardized uptake value, 18F-FDG, fluorodeoxyglucose (18F), 3Y-OS, three-year overall survival

## Abstract

Intravascular large B-cell lymphoma (IVLBCL) is a rare and aggressive subtype of extranodal non-Hodgkin lymphoma (NHL) that typically involves the central nervous system (CNS) and skin in its classic (Western) variant and frequently presents with systemic B-symptoms. We present a rare case of IVLBCL in a 59-year-old female with cutaneous-only involvement, no B-symptoms, and negative PET-CT. The patient’s initial presentation mimicked inflammatory conditions, contributing to diagnostic delay. Deep skin biopsy was crucial and confirmed the diagnosis of IVLBCL. PET-CT was non-revealing, showing limitations in cutaneous-dominant disease. The patient underwent R-CHOP treatment along with CNS prophylaxis, achieving complete remission with no relapse at follow-up. This case emphasizes the importance of high suspicion and early biopsy in atypical cutaneous presentations and the need for personalized strategies.

## 1 Introduction

Intravascular large B-cell lymphoma (IVLBCL) is a rare and aggressive extranodal subtype of Non-Hodgkin lymphoma (NHL), defined by the proliferation of malignant B lymphocytes within small and medium-sized blood vessels, therefore possibly affecting any organ. The condition affects both genders equally, typically between 60–70 years of age (median of 72 years), with an estimated incidence of fewer than 0.5 cases per million. The pathogenesis is not fully understood. IVLBCL cells express molecules involved in cell migration and adhesion to the endothelium, but lack those involved in extravasation, which is a differentiating factor to Diffuse large cell B-lymphoma (DLBCL) [1]. IVLBCL is clinically classified into two major variants: the classic (Western) form, characterized by CNS and cutaneous involvement, and the hemophagocytic (Asian) form, which more frequently affects the bone marrow, liver, and spleen. B-symptoms, elevated inflammatory markers, and cytopenias are commonly seen in both variants [1,2]. A third, cutaneous-only variant has been described, typically occurring in younger females, with isolated skin manifestations, normal blood counts, and a relatively favorable prognosis [[1], [2], [3]]. Diagnosis is often difficult due to the disease’s non-specific presentation and histological confinement to the vasculature. PET-CT may aid in staging but has limitations – approximately one-third of IVLBCL lesions may be FDG non-avid [4]. Deep skin biopsy remains key, particularly when skin findings are prominent [1,5]. Skin presentations themselves are often cumbersome to properly diagnose with a broad range of differential diagnosis [2]. Treatment includes prompt initiation of R-CHOP therapy, which has an overall response rate of > 60%, and 3Y-OS of > 30%. While outcomes improved, a significant number of patients still relapse [1,2]. The most used prognostic model is The International Prognostic Index (IPI), along with some newer markers, such as serum albumin, Ki-67 index, sIL-2R, and comorbidity status [2]. We present a rare cutaneous-only IVLBCL case without systemic B-symptoms or PET-CT positivity.

## 2 Case report

The patient was a 59-year-old woman with no significant past medical history apart from a diclofenac-induced urticaria and a family history of kidney cancer. In May 2023, she was diagnosed with mononucleosis, with ultrasound revealing splenomegaly (15 × 6 cm). In November 2023, she developed painful, progressively enlarging nodular lesions on the medial thighs, accompanied by burning sensations, but no B-symptoms such as fever, weight loss, or night sweats. From December 2023 to February 2024, she was under hematologic follow-up for hypersplenism, sideropenic anemia, and monocytosis, which was presumed to be post-infectious.

Borrelia serology was positive in January 2024, and she received azithromycin. While skin findings worsened, she was hospitalized at the dermatology department in February of 2024 with suspected panniculitis. Examination revealed bilateral, violaceous efflorescences accompanied by painful subcutaneous nodules on the medial thighs. Oral and topical corticosteroids led to improvement. Key laboratory values for pre-oncohematologic treatment are stated in Table 1 below.

**Table 1:** Selected laboratory values during the work-up phase (12/2023), throughout treatment, and after treatment completion.

DATE	Leukocytes (WBC) (x 10⁹/L)	Hemoglobin (HGB) (g/dL)	Platelets (PLT) (x 10⁹/L)	β_2_-Microglobulin (mg/L)
12/2023	6.45	9.4	151	4.87
06/2024	24.0	10.0	251	3.4
07/2024	6.7	11.0	291	3.2
08/2024	7.4	11.6	363	3.6
11/2024	6.9	12.9	276	2.7

As part of the work-up, a deep skin biopsy of the left thigh was performed, revealing features consistent with IVLBCL. Involvement of richly vascularized subcutaneous fibrolipomatous tissue was reported, with small- to medium-sized vessels exhibiting perivascular lymphoid infiltrates, as well as exclusively intravascular medium- to large-sized blasts, occasionally accompanied by hyaline thrombi. Immunophenotyping revealed positivity for B-cell markers (CD20, CD45, CD79a, PAX5), BCL2, BCL6, and equivocal partial CD10 expression. MUM1 was positive, with a high Ki-67 index (>90%). Tumor cells were negative for CD3, CD5, TdT, cyclin D1, and CD138, while c-MYC was partially and variably positive. Accompanying reactive perivascular infiltrate consisted predominantly of CD3+/CD5+ T cells, with fewer B cells and CD138+ plasma cells. No hemophagocytosis was observed. Selected histopathological features are shown in Fig. 1.

**Fig. 1:**
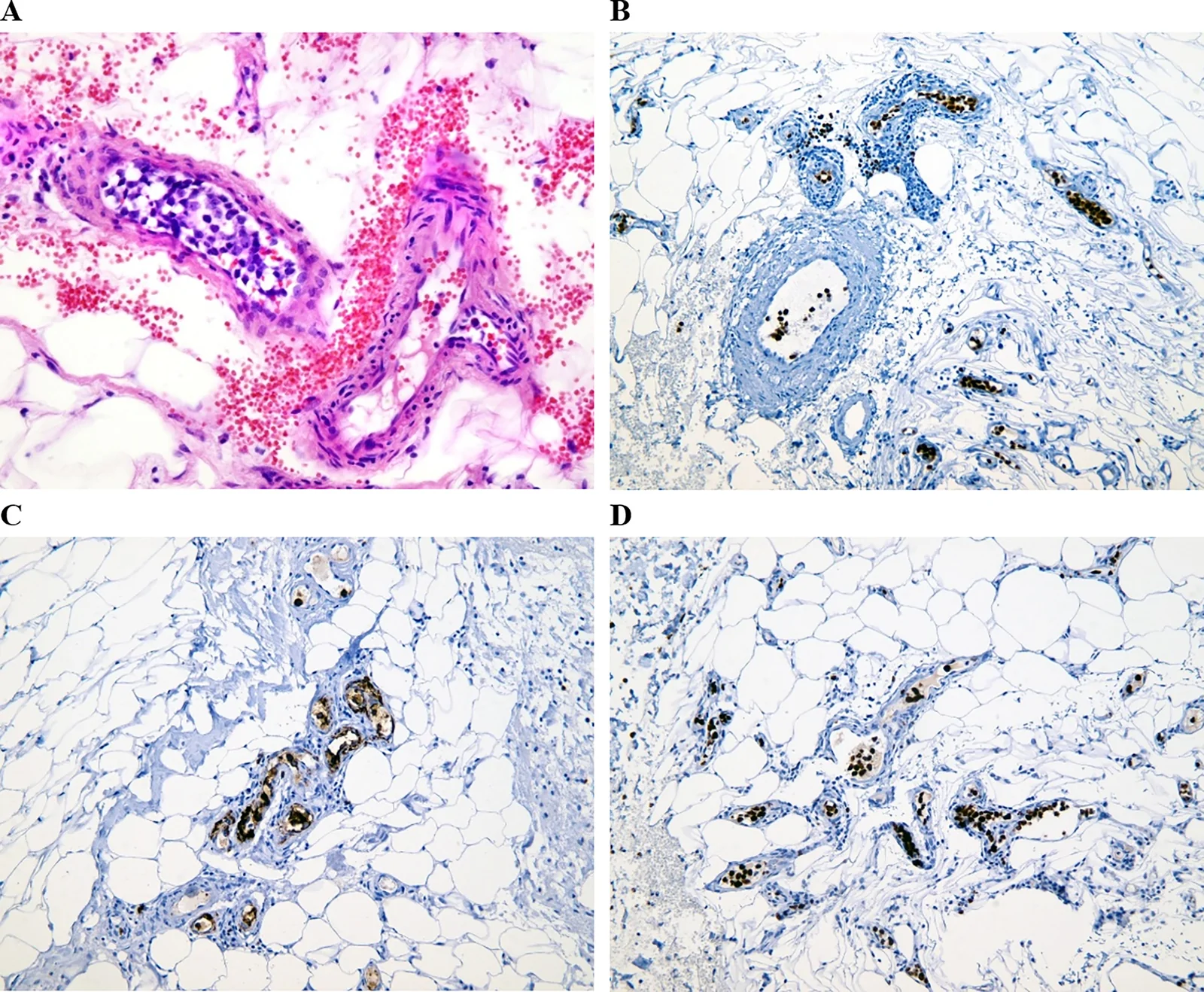
Histopathological and immunohistochemical features of IVLBCL. A: H&E staining demonstrating atypical B-lymphocytes in the lumen of subcutaneous postcapillary venules and capillaries, x400. B: PAX5 immunohistochemistry showing nuclear positivity in intravascular neoplastic B cells, confirming B-cell lineage, x200. C: CD20 immunohistochemistry demonstrating membranous positivity in intravascular neoplastic B cells, x200. D: Ki-67 immunohistochemistry revealing an elevated proliferative index (>90%) in the neoplastic cells, x200.

FISH analysis showed no MYC (8q24) rearrangement. Bone marrow examination was unremarkable. A second deep skin biopsy from the right thigh (May 2024) confirmed prior findings. Lumbar puncture and intrathecal chemotherapy were administered with CSF cytology being negative. Baseline CT revealed hepatosplenomegaly, small hypodense liver lesions, breast nodules, and minor pulmonary and subpleural changes. PET-CT (March 2024) confirmed hepatosplenomegaly but showed no hypermetabolic lesions in the lungs, liver, or breasts. Fibrotic changes in the medial thighs were noted, without metabolic activity.

The patient completed six cycles of R-CHOP with two additional rituximab infusions by November 2024. Treatment was well tolerated, leading to hematologic normalization (as seen in Table 1), resolution of skin lesions, regression of splenomegaly and a negative PET-CT finding. No relapse has occurred to date.

## 3 Discussion

IVLBCL remains a diagnostic challenge due to its atypical presentation and frequent absence of lymphadenopathy or mass lesions. In this case, the disease presented with exclusive cutaneous involvement and no B-symptoms, initially mimicking inflammatory conditions. Dermatologic findings were suspected to be panniculitis and improved with steroids.

This skin-only presentation, more common in younger female patients, has been associated with better prognosis, consistent with the patient’s favorable outcome, and it is believed to be attributable to earlier diagnosis, lower stage and better performance status [1,2]. Histopathological diagnosis via deep skin biopsy with sufficient depth to capture affected blood vessels was critical in identifying the disease. Multiple biopsies may enhance diagnostic yield, and some authors advocate for at least three from both affected and unaffected areas [5].

In our case, PET-CT was not revealing, limited by non-parenchymal presentation. The patient responded well to R-CHOP chemotherapy, which is consistent with the literature [1,2]. The question of CNS prophylaxis in this case remains controversial. The R-CHOP regimen does not cross the blood–brain barrier [1,2], upfront neuroimaging may not always detect disease spread [6] and some studies report CNS involvement in up to 25% of cases during follow-up [7]. Lumbar puncture is therefore warranted, and while false negatives are rare [1], the decision about CNS prophylaxis must be individualized. In our case, we decided to proceed with CNS prophylaxis. Although the patient is currently in remission, the potential for relapse remains, particularly as follow-up duration has been just under two years. Continued monitoring is therefore needed to fully assess long-term outcomes. Cutaneous-only IVLBCL is rare, and cases without B-symptoms or PET-CT findings are even less frequently reported.

Compared to similar studies, our report offers several distinguishing features. Maiolo *et al.* [8] described a 77-year-old woman with panniculitis-like presentation without initial clear B-symptoms, but subsequent PET findings were positive and final diagnosis made after pathology revision. B-symptoms developed subsequently as well. Bayçelebi *et al.* [9] reported a 46-year-old woman with panniculitis-like presentation and without imaging findings. Diagnosis was made via biopsy reevaluation only after clinical worsening despite treatment. Park *et al.* [10] described a 62-year-old woman with cutaneous lesions, no systemic symptoms, and PET-CT positivity. All three patients responded to R-CHOP, with the case reported by Park *et al.* [10] complicated by rituximab-related lung toxicity.

An additional finding in our patient was positive Borrelia serology during the initial diagnostic work-up. *Borrelia burgdorferi* infection has been implicated mainly in the pathogenesis of primary cutaneous B-cell lymphomas, with supporting evidence including detection of Borrelia DNA in lymphoma tissue [11]. However, a similar association with IVLBCL has not been established. Borrelia specific testing has appeared in some IVLBCL case studies during workups, without establishing an associative relationship [12]. Furthermore, a nationwide cohort study found no increased risk of hematologic malignancies among Borrelia-seropositive individuals compared with seronegative controls [13]. Anti-Borrelia antibodies may persist for many years following infection and do not necessarily indicate active disease [14]. Therefore, the positive Borrelia serology in our patient should be regarded as a finding of uncertain clinical significance rather than evidence of a causal relationship with IVLBCL.

The presented cases along with ours demonstrate diagnostic complexity and the need for a multidisciplinary approach, along with the essential role of deep biopsy. Our case is distinct in being without B-symptoms, PET-CT negative despite confirmed cutaneous disease, steroid-responsive at onset, featuring extensive immunophenotyping and CNS-directed management.

## 4 Conclusion

This case highlights the importance of considering IVLBCL in patients presenting with unexplained panniculitis-like cutaneous lesions, even in the absence of systemic B-symptoms or positive PET-CT findings. Early deep skin biopsy, multidisciplinary assessment, and prompt initiation of appropriate treatment are essential for timely diagnosis and management. Although cutaneous-only IVLBCL may be associated with a more favorable prognosis, continued follow-up remains necessary because of the potential for relapse. Further studies are needed to improve diagnostic strategies and prognostic assessment in this rare lymphoma subtype.

## Ethics declaration

Written informed consent to take part in the study and to publish the article has been obtained from all participants or their legal representatives. The privacy rights of participants have been observed.

This study was performed in compliance with relevant laws, regulatory frameworks and guidelines where the research took place. Ethics committee approval was not required under relevant laws and institutional guidelines. Ethics committee approval was not required for this single-patient case report in accordance with applicable institutional policies.

This research follows the CARE guidelines and the CARE checklist.

## CRediT authorship contribution statement

**Juliana Gabzdilova:** Writing – review & editing, Writing – original draft, Conceptualization. **Atila David Homolya:** Writing – review & editing, Writing – original draft, Investigation. **Stefan Raffac:** Writing – original draft, Supervision. **Frantisek Gmitter:** Supervision. **Tomas Guman:** Supervision, Conceptualization. **Viktoria Hazikova:** Writing – review & editing, Investigation. **Martina Maria Raffacova:** Supervision, Investigation. **Filip Breznen:** Supervision, Investigation.

## Declaration of competing interest

The authors declare no competing financial interests or personal relationships that could influence the work reported in this manuscript.
